# A flow-cytometry-based assay to assess the cytolytic activity against tumor cells by combination of mouse MAIT cells and natural killer cells

**DOI:** 10.1016/j.xpro.2023.102620

**Published:** 2023-10-27

**Authors:** Chie Sugimoto, Hiroyoshi Fujita, Hiroshi Wakao

**Affiliations:** 1 Host Defense Division, Research Center for Advanced Medical Science, Dokkyo Medical University, Mibu, Tochigi 321-0293, Japan

**Keywords:** Cell Biology, Cell Culture, Cell Isolation, Flow Cytometry/Mass Cytometry, Cancer, Immunology, Model Organisms, Stem Cells, Cell Differentiation

## Abstract

Mucosal-associated invariant T (MAIT) cells are innate-like T cells responsible for mucosal immunity in the respiratory and intestinal tracts. Here we present a flow-cytometry-based assay to measure the cytolytic activity of murine MAIT cells and natural killer (NK) cells. We describe steps for differentiating MAIT-like cells from the induced pluripotent stem cells prepared from MAIT cells (reMAIT cells), NK cell isolation, co-culture with target tumor cells, and staining to distinguish dead cells from live cells.

For complete details on the use and execution of this protocol, please refer to Sugimoto et al. (2022).^1^

## Highlights

• Differentiation of murine MAIT-like cells from iPSCs (reMAIT cells)
• Isolation of murine NK cells from the spleen
• Preparation of CFSE-labeled cancer cells
• Flow cytometric assay for the cytolytic activity against cancer cells with Zombie stain

## Abstract (editor highlights)

Publisher’s note: Undertaking any experimental protocol requires adherence to local institutional guidelines for laboratory safety and ethics.

## Graphical abstract

**Figure:**
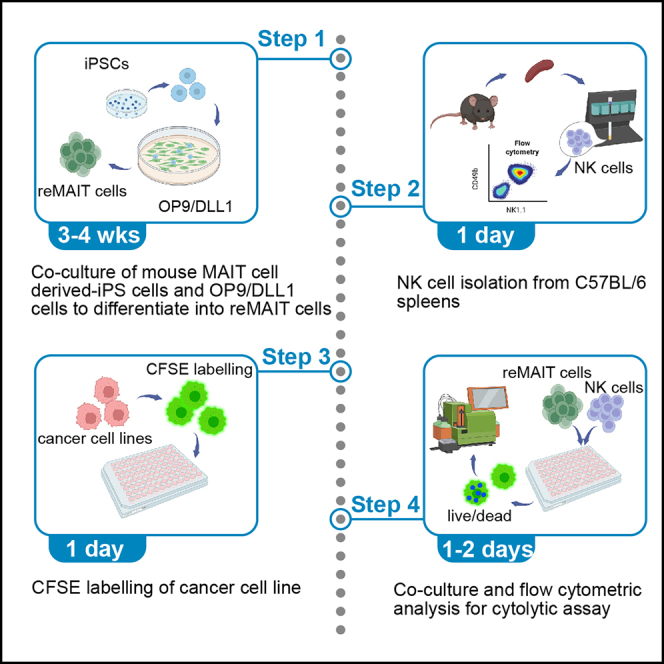

## Before you begin

The protocol below describes the specific steps in a cytolytic assay for redifferentiated mouse MAIT-like cells from iPS Cells (iPSCs) (reMAIT cells). While human MAIT cells comprise about 0.5%–10% of peripheral blood T cells, less than 0.05% represent MAIT cells in laboratory mice. To investigate the function of MAIT cells in mouse disease models, we obtain a large amount of reMAIT cells from iPSCs. For this purpose, the iPSCs must possess rearranged T cell receptor (TCR) loci specific for MAIT cells. Therefore, the iPSCs must be established from MAIT cells.

Another way to obtain mouse MAIT cells is to isolate them from the tissues of laboratory mice with increased number of MAIT cells. Such mice may include MAIT-TCR transgenic mice,^2^ B6-Cast mice,^3^ wild-type mice exposed to the vaccine strains of bacteria or MAIT cell ligands such as vitamin B2 metabolites,^4^ and Vα19 or Vβ8 mice generated via chimeric mice from MAIT-iPSC, which we recently reported.^5^^,^^6^ When using MR1-tetramers that specifically detect MAIT cells, it should be recalled that MAIT cells are activated during the purification process.^1^ Therefore, when interpreting results using MAIT cells purified with the MR1 tetramer, one must be cautious about whether the *in vitro* results truly reflect the *in vivo* features.

### Institutional permissions

The ethical approval for animal experiments is required prior to beginning this procedure. All mouse experiments in this protocol were performed with approval from the Institutional Animal Care and Use Committee of Dokkyo Medical University (Approval no. 1215).

### Preparation of culture media, cytokines, and gelatinized plates

**Timing: ∼2 days**

1. Prepare the culture media, reagents and cytokines as described in materials and equipment section below.
2. Prepare gelatinized 12-well plates and 10-cm cell culture dishes.
  a. Add 1 mL and 6 mL of 0.1% gelatin (stored at 20°C–25°C up to 3 months) into each well of 12-well plates and each 10-cm dish, respectively.
  b. Incubate plates and dishes in a 37°C CO_2_ incubator for at least 30 min for gelatin coating.
  c. Plates and dishes can be stored in an incubator for about 10 days. Gelatin solution should not be allowed to dry completely.

### Preparation and maintenance of OP9/delta-like 1(DLL1) cells

**Timing: 1**∼**2 weeks (Prepare before starting reMAIT cell differentiation)**

**Timing: 4–6 days (for step 4)**

3. Prepare and maintain OP9/DLL1 culture.
  a. Pre-warm 10 mL of OP9 medium (Stored at 4°C up to 1 month) in a 15-mL conical tube in a 37°C water bath.
  b. Quick-thaw frozen OP9/DLL1 cells in a 37°C water bath and transfer them to a pre-warmed 15-mL conical tube containing the medium in step 3a.
  c. Centrifuge the cells at 400 × *g* for 5 min. Remove the supernatant and suspend the cells in 10 mL of OP9 medium.
  d. Transfer the cell suspension (1.0–1.2 × 10^6^ cells) to a 10-cm culture dish and spread the cells evenly throughout the dish by cross-shaking.
  e. Incubate in a 5% CO_2_ incubator at 37°C until the cells reach nearly confluent.
  f. Passage nearly confluent OP9/DLL1 monolayer with trypsin.
    i. Remove the medium and wash the monolayer with 5 mL of PBS (20°C–25°C) twice.
    ii. Add 0.5 mL of 0.25% Trypsin-1 mM EDTA (Stored at 4°C up to 2 weeks. Use at 20°C–25°C) and spread well.
    iii. Incubate the dish in a 5% CO_2_ incubator at 37°C for 3–5 min.
    iv. Add 4 mL of OP9 medium and suspend well to make single cell suspension.
    v. Transfer the cell suspension to a 15-mL conical tube and centrifuge at 400 × *g* for 5 min.
    vi. Remove the supernatant and suspend the cells in an appropriate amount of OP9 medium.
    vii. Passage the cells to new 10-cm culture dishes at a ratio 1:3 to 1:8. Determine the ratio so that the same confluency is achieved in every 3 days.
    viii. Incubate the dishes in the Incubator.
  g. Repeat e. and f. as needed to maintain the cells.
4. Prepare co-culture plates for reMAIT cell differentiation.
  a. Trypsinize nearly-confluent OP9/DLL1 monolayer and make cell suspension as described in Step 3-f-i to -vi.
  b. Remove gelatinized 10-cm culture dishes from the incubator and aspirate off the gelatin solution.
  c. Split OP9/DLL1 cells into gelatinized dishes at the same passage ratio as maintained cells with OP9 medium.
  d. Incubate in the CO_2_ incubator for 4–6 days. Replace half of OP9 medium every other day.

**CRITICAL:** Be careful not to overgrow OP9/DLL1 cells (overconfluent) during the maintenance. OP9/DLL1 cells tend to increase in growth rate along with the number of passages; if it becomes confluent within 3 days at 1:8, a new stock should be thawed. Therefore, it is recommended making stocks in the early passages. We usually freeze the cells that are 80% confluent in 10-cm culture dish per cryovial using a common cell-freezing medium (we use BAMBANKER). In that case, you can start passaging the day after the frozen cells are thawed.

### Preparation and maintenance of mouse MAIT cell-derived iPSCs

**Timing: 1 week (Prepare before starting reMAIT cell differentiation)**

5. Prepare mouse embryonic fibroblast (MEF) feeder cells.
  a. Pre-warm 10 mL of MEF medium in a 15-mL conical tube in a 37°C water bath.
  b. Quick-thaw a frozen vial (1.25 × 10^6^ cells/vial) of mitomycin C-treated MEF in a 37°C water bath and transfer them to a pre-warmed 15-mL conical tube containing MEF medium in step 5-a.
  c. Centrifuge the cells at 400 × *g* for 5 min.
  d. Remove the supernatant and suspend the cells in 12 mL of MEF medium (stored at 4°C up to 1 month).
  e. Remove a gelatinized 12-well plate from the incubator and aspirate off the gelatin solution.
  f. Spread 1 mL of MEF suspension per well.
  g. Incubate in the CO_2_ incubator for 2 days.
6. Prepare and maintain mouse MAIT-iPSCs
  a. Pre-warm 10 mL of MEF medium in a 15-mL conical tube in a 37°C water bath.
  b. Quick-thaw a frozen vial of iPSCs in a 37°C water bath and transfer them to a pre-warmed 15-mL conical tube containing MEF medium in step 6-a.
  c. Centrifuge the cells at 400 × *g* for 5 min.
  d. Remove the supernatant and suspend the cells in 1 mL of mES medium (stored at 4°C up to 2 weeks).
  e. Remove the medium from a well containing MEF feeder cells as prepared in step 5.
  f. Seed iPSC suspension into a well containing MEF feeder cells.
  g. Incubate in the CO_2_ incubator until iPSCs grow to 70%–80% confluency. During the incubation, change half of the medium with mES medium every day.
  h. Once iPSCs grow to 70%–80% confluency, they are passaged on. Therefore, MEF feeder cells must be prepared in advance according to step 5.
  i. Wash iPSCs with 1 mL of PBS twice.
  j. Add 200 μL of 0.25% Trypsin-1 mM EDTA per well and spread evenly, and incubate in a 37°C incubator for 2–3 min.
  k. Check whether the cells are completely detached. Add 1 mL of MEF medium, pipetting well to make single cell suspension.
  l. Determine the cell number. 1.2 × 10^5^ cells and 6 × 10^4^ cells are used for passages at 3-day and 4-day intervals, respectively.
  m. Remove the medium from a new well filled with MEF feeder cells and add 1 mL of mES medium.
  n. Seed the appropriate amount of iPSC suspension as shown in step 6-l into a well filled with MEF feeder cells.
  o. Incubate in the CO_2_ incubator until iPSCs grow to 70%–80% confluency. During the incubation, change half of the medium with mES medium every day.

**CRITICAL:** MMC-treated MEFs often do not adhere to plates or dishes. This problem can be overcome by adding 2-mercaptoethanol to the medium, as in the MEF medium shown in this protocol.

***Alternatives:*** We use commercial MEFs and treat them with MMC, but MEFs can also be prepared from 13-14 days-embryos of mice (there is a protocol elsewhere) and MMC-treated MEFs are commercially available.

### Culture of the cancer cell lines

**Timing: 1 week (Prepare before starting cytolysis assay)**

YAC-1, a lymphoma induced by inoculation of Moloney leukemia virus into a newborn A/Sn mouse, is known as a good target cell line for NK cells. Lewis lung carcinoma (LLC) is a cell line established from spontaneous lung carcinoma in C57BL/6. LLC shows high metastatic potential *in vivo* and is resistant to various anti-cancer drugs and to cytolytic T cells. Although LLC expresses low levels of MR1, which presents antigens to MAIT cells, it is not known whether MAIT cells recognize LLC via the MR1-TCR axis. Regardless of the mode of tumor cell recognition, it is important to note that reMAIT cells exhibit cytolytic activity against these tumor cell lines.

7. YAC-1 culture.
  a. Pre-warm 10 mL of cR10 medium (stored at 4°C up to 1 month) in a 15-mL conical tube in a 37°C water bath.
  b. Quick-thaw a frozen vial of YAC-1 in a 37°C water bath and transfer them to a pre-warmed 15-mL conical tube containing cR10 medium in step 7-a.
  c. Centrifuge the cells at 400 × *g* for 5 min.
  d. Remove the supernatant and suspend the cells in 5 mL of cR10 medium.
  e. Transfer the cell suspension into a 25-cm^2^ culture flask.
  f. Incubate the flask upright in the CO_2_ incubator.
  g. Passage at 1:20 twice a week.
8. LLC culture.
  a. Pre-warm 10 mL of 8%FBS/DMEM medium (stored at 4°C up to 1 month) in a 15-mL conical tube in a 37°C water bath.
  b. Quick-thaw a frozen vial of LLC in a 37°C water bath and transfer them to a pre-warmed 15-mL conical tube containing 8%FBS/DMEM medium in step 8-a.
  c. Centrifuge the cells at 400 × *g* for 5 min.
  d. Remove the supernatant and suspend the cells in 10 mL of 8%FBS/DMEM medium.
  e. Spread the cell suspension in a 10-cm culture dish.
  f. Incubate in the CO_2_ incubator.
  g. Passage at 1:4 twice a week.

***Note:*** YAC1 is a good proliferating cell line and has no problems for maintenance in suspension culture. LLC is difficult to grow if the cell density is too low. It is thus recommended to passage the cells at a higher concentration or to save the LLC-cultured medium and add it as a conditioned medium during passages (See troubleshootingproblem 3).

## Key resources table

**Table:** 

REAGENT or RESOURCE	SOURCE	IDENTIFIER
**Antibodies**		

Anti-mouse CD49b-PE (DX5, 1:100 dilution in 50 μL reaction)	BioLegend	Cat# 108907; RRID:AB_313414
Anti-mouse NK1.1-FITC (PK136, 1:100 dilution in 50 μL reaction)	BioLegend	Cat# 108706, RRID:AB_313393
Anti-mouse TCRβ-PE/Cy7 (H57-597, 1:100 dilution in 50 μL reaction)	BioLegend	Cat# 109222, RRID:AB_893625

**Biological samples**		

Mouse embryonic fibroblast (MEF)	Oriental Yeast	Cat# KBL9284600
Fetal bovine serum (FBS)	Gibco	

**Chemicals, peptides, and recombinant proteins**		

Mouse MR1 5-OP-RU tetramer, APC-labeled (1:1000 dilution in 50 μL reaction)	NIH Tetramer Core Facility	N/A
Mouse MR1 6-FP tetramer, APC-labeled (1:1000 dilution in 50 μL reaction)	NIH Tetramer Core Facility	N/A
Mitomycin C solution (1 mg/mL)	Nacalai Tesque	Cat# 20898-21
NEAA	FUJIFILM Wako	Cat# 139-15651
L-glutamine	Nacalai Tesque	Cat# 16948-04
penicillin/streptomycin	Lonza	Cat# 09–757F
2-Mercaptoethanol	FUJIFILM Wako	Cat# 133-06864
Mouse LIF	FUJIFILM Wako	Cat# 195-16053
CHIR99021	FUJIFILM Wako	Cat# 034-23103
PD0325901	FUJIFILM Wako	Cat# 168-25293
αMEM	Thermo Fisher Scientific	Cat# 11900024
Recombinant mouse interleukin 7	BioLegend	Cat# 577802
Recombinant human FLT3 ligand	BioLegend	Cat# 550606
RPMI1640	Nacalai Tesque	Cat# 30264-56
DMEM	Nacalai Tesque	Car# 08458-16
HBSS	FUJIFILM Wako	Cat# 084-08345
HEPES	FUJIFILM Wako	Cat# 047861
Percoll	Cytiva	Cat# 17089101
7-AAD (7-aminoactinomycin D)	MedChemExpress	Cat# HY-D1020
Gelatin	FUJIFILM Wako	Cat# 077-03155
Bovine serum albumin fraction V (BSA)	FUJIFILM Wako	Cat# 011-27055
0.25% Trypsin-1 mM EDTA	FUJIFILM Wako	Cat# 201-16945
10× PBS	FUJIFILM Wako	Cat# 048-29805
BANBANKER	GC LYMPHOTEC	N/A

**Critical commercial assays**		

MojoSort mouse NK isolation kit	BioLegend	Cat# 480050
Vybrant CFDA SE cell tracer kit	Thermo Fisher Scientific	Cat# V12883
Zombie Violet Flexible Viability kit	BioLegend	Cat# 423113

**Experimental models: Cell lines**		

Murine MAIT cell-derived iPSCs (established from male C57BL/6NJcl)	Dr. Hiroshi Wakao (Dokkyo Medical University)^1^	N/A
OP9 -DLL1 cell	Dr. Hiroshi Kawamoto (Kyoto University)^7^	N/A
YAC-1 cell	Riken Bioresource Center	Cat# RCB1165; RRID: CVCL_2244
Lewis lung carcinoma (LLC)	Riken Bioresource Center	Cat# RCB0558; RRID: CVCL_4358

**Experimental models: Organisms/strains**		

C57BL/6NJcl female and male 8–12 week old	CLEA Japan	N/A

**Software and algorithms**		

FlowJo software v9 and v10	BD Bioscience	N/A

## Materials and equipment

**Table:** 0.1% Gelatin

Reagent	Final concentration	Amount
MilliQ water	-	500 mL
Gelatin powder	0.1%	0.5 g
**Total**	**N/A**	**500 mL**

**Note:** Autoclave at 121°C for 20 min. Store at 20°C–25°C (Stable at 20°C–25°C for at least 3 months. For longer storage, 4°C is recommended.).

**Table:** MEF medium

Reagent	Final concentration	Amount
DMEM	-	500 mL
FBS	7.4%	40 mL
0.1 M 2-mercaptoethanol	0.1 mM	500 μL
penicillin/streptomycin (200×)	100 U penicillin/100 μg/mL streptomycin	2.5 mL
**Total**	**N/A**	**543 mL**

**Note:** Store at 4°C up to 1 month. 0.1 M 2-mercaptoethanol: add 100 μL of 2-mercaptoethanol in 14.2 mL of PBS.

Storage conditions for 0.1 M 2-mercaptoethanol: Make aliquots and store at −20°C.

**Table:** mES medium

Reagent	Final concentration	Amount
DMEM	-	42.5 mL
FBS	15%	7.5 mL
NEAA (100×)	1×	500 μL
200 mM L-glutamine (100×)	1×	500 μL
Penicillin/streptomycin (200×)	100 U penicillin/100 μg/mL streptomycin	250 μL
mouse LIF (10^6^ units/mL)	10^3^ units/mL	50 μL
0.1 M 2-mercaptoethanol	0.1 mM	50 μL
15 mM CHIR99021	3 μM	10 μL
10 mM PD0325901	1 μM	5 μL
**Total**	**N/A**	**50 mL**

**Note:** Store at 4°C up to 2 weeks.

• 0.1 M 2-mercaptoethanol: add 100 μL of 2-mercaptoethanol in 14.2 mL of PBS.

Note on storage conditions: Make aliquots and store at −20°C.

• 15 mM CHIR99021: add 716 μL of DMSO into a vial containing 5 mg of CHIR99021.

Note on storage conditions: Make aliquots and store at −20°C.

• 10 mM PD0325901: add 1037 μL of DMSO into a vial containing 5 mg PD0325901.

Note on storage conditions: Make aliquots and store at −20°C.

**Table:** Complete R10 (cR10)

Reagent	Final concentration	Amount
RPMI1640	-	500 mL
FBS	9%	50 mL
1 M HEPES (pH7.0)	10 mM	5 mL
0.1 M 2-mercaptoethanol	0.1 mM	500 μL
Penicillin/streptomycin (200×)	100 U penicillin/100 μg/mL streptomycin	2.5 mL
**Total**	**N/A**	**558 mL**

**Note:** Store at 4°C up to 1 month.

• 0.1 M 2-mercaptoethanol: add 100 μL of 2-mercaptoethanol in 14.2 mL of PBS.

Note on storage conditions: Make aliquots and store at −20°C.

**Table:** 8% FBS/DMEM

Reagent	Final concentration	Amount
DMEM	-	500 mL
FBS	7.3%	40 mL
Penicillin/streptomycin (200×)	100 U penicillin/100 μg/mL streptomycin	2.5 mL
**Total**	**N/A**	**542.5 mL**

**Note:** Store at 4°C up to 1 month.

**Table:** αMEM

Reagent	Final concentration	Amount
αMEM powder	-	21.6 g
Sodium bicarbonate	-	4.4 g
MilliQ water	-	2 L
**Total**	**N/A**	**2 L**

**Note:** Store at 4°C up to 1 month.

• Prepare 21.6 g of αMEM powder and 4.4 g of sodium bicarbonate in a container.
• Add 2 L MilliQ water.
• Dissolve with a magnetic stirrer.
• Sterilize the medium using a bottle top filter (0.22 μm).

**Table:** OP9 medium

Reagent	Final concentration	Amount
αMEM	-	800 mL
FBS	16.7%	160 mL
**Total**	**N/A**	**960 mL**

**Note:** Store at 4°C up to 1 month.

**Table:** Differentiation medium

Reagent	Final concentration	Amount
αMEM	-	800 mL
FBS	9.1%	80 mL
**Total**	**N/A**	**880 mL**

**Note:** Store at 4°C up to 1 month.

**Table:** reMAIT medium

Reagent	Final concentration	Amount
OP9 medium	-	100 mL
100 μg/mL human FLT3-L	5 ng/mL	5 μL
10 μg/mL mouse IL-7	1 ng/mL	10 μL
Penicillin/streptomycin (200×)	100 U penicillin/100 μg/mL streptomycin	500 μL
**Total**	**N/A**	**100 mL**

**Note:** Store at 4°C up to 10 days.

• 100 μg/mL human FLT3-L: dissolve 100 μg human FLT3-L in 1000 μL of PBS containing 1% BSA.

Note on storage conditions: Make aliquots and store at −80°C.

• 10 μg/mL mouse IL-7: dissolve 10 μg mouse IL-7 in 1000 μL of PBS containing 1% BSA.

Note on storage conditions: Make aliquots and store at −80°C.

**Table:** MACS buffer

Reagent	Final concentration	Amount
PBS	-	238.5 mL
10% BSA	0.5%	12.5 mL
0.5 M EDTA	2 mM	1 mL
**Total**	**N/A**	**250 mL**

**Note:** Store at 4°C up to 3 months.

**Table:** FACS buffer

Reagent	Final concentration	Amount
PBS	-	500 mL
FBS	2%	10 mL
10% Sodium azide	0.1%	5 mL
**Total**	**N/A**	**515 mL**

**Note:** Store at 4°C up to 3 months.

**Table:** 67% Ficoll

Reagent	Final concentration	Amount
Isotonic Percoll	60%	67 mL
HBSS	-	33 mL
**Total**	**N/A**	**100 mL**

**Note:** Store at 4°C up to 3 months.

• Isotonic Percoll: mix 90 mL of Percoll with 10 mL of 10× PBS.

Note on storage conditions: Store at 4°C.

**Table:** 40% Ficoll

Reagent	Final concentration	Amount
Isotonic Percoll	36%	40 mL
HBSS	-	60 mL
**Total**	**N/A**	**100 mL**

**Note:** Store at 4°C up to 3 months.

• Isotonic Percoll: mix 90 mL of Percoll with 10 mL of 10× PBS.

Note on storage conditions: Store at 4°C.

**Table:** 7-AAD

Reagent	Final concentration	Amount
7-AAD	25 μg/mL	1 mg
PBS	-	40 mL
**Total**	**N/A**	**40 mL**

**Note:** Store at 4°C up to 6 months.

## Step-by-step method details

### Differentiation of reMAIT cells from MAIT cell-derived iPSCs

**Timing:** ∼**3 weeks**

Here, we describe each step for differentiating MAIT-like cells (reMAIT cells) from MAIT cell-derived iPSCs (MAIT-iPSCs), partially modifying a previous protocol on T cell differentiation from mouse ES cells using OP9/DLL1 cells.^8^

1. iPSC culture on OP9/DLL1 (Day 0).
  a. Take day-4 to -5 OP9/DLL1 dishes (see step 4 in before you begin, Figure 1A) from the incubator and replace the medium with 9 mL of Differentiation medium (stored at 4°C up to 1 month).

**Figure 1:**
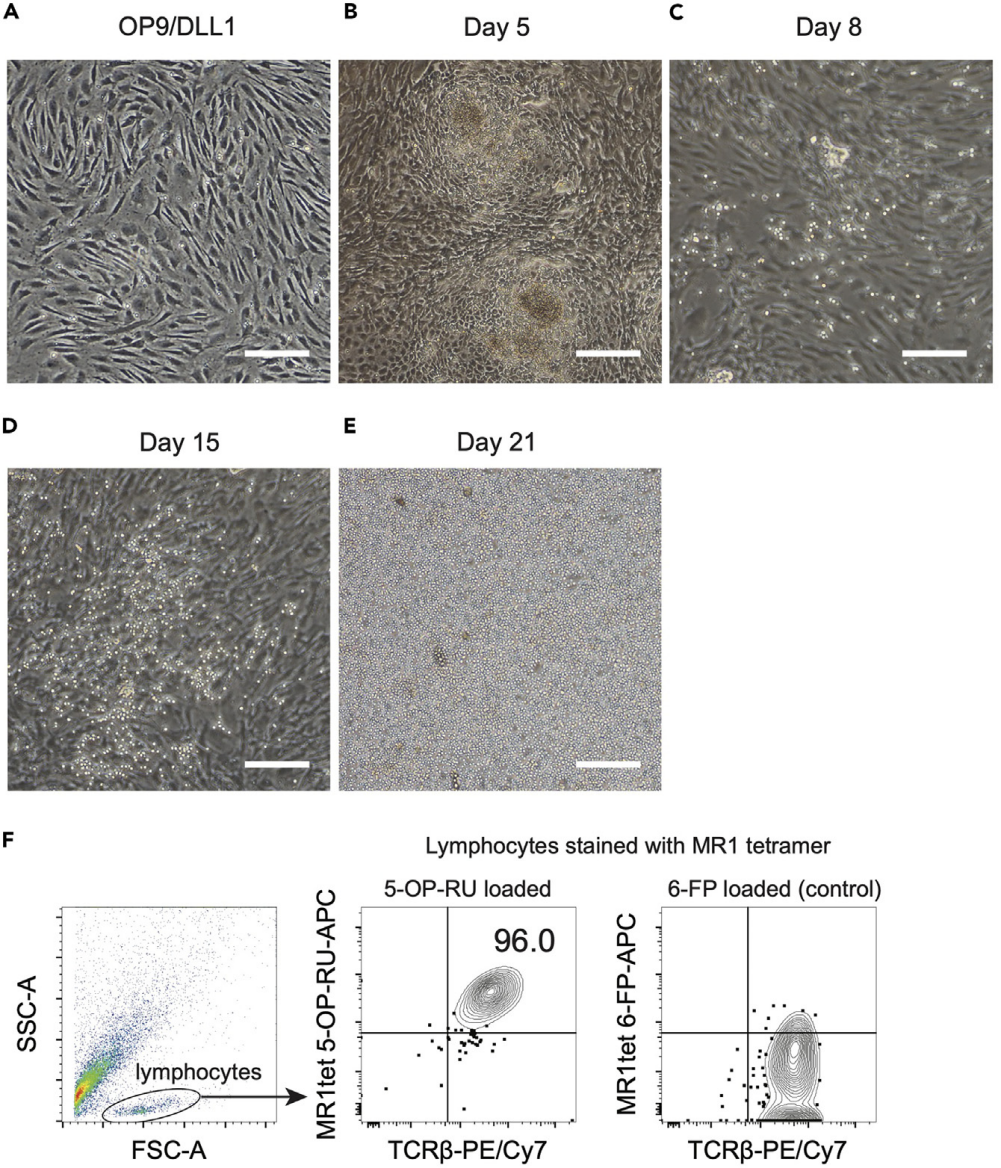
reMAIT cell differentiation from MAIT-iPSCs on OP9/DLL1 monolayer (A) OP9/DLL1 monolayer on day 4 after seeding on gelatin-coated dish. This picture shows optimal confluency for initiation of reMAIT cell differentiation. (B) After the medium change on day 3, a rapid morphological change is observed, and by day 5, mesodermal cell masses with raised center are formed. (C) Mesodermal cells differentiate into lymphoblasts on new OP9/DLL1 in the presence of FLT3-L (Day 8). Lymphoblasts have a grape cluster-like morphology. (D) Lymphoblasts differentiate into reMAIT cells and reMAIT cells are expanding in the presence of FLT3-L and IL-7. (E) The proliferating reMAIT cells can be transferred onto a new OP9/DLL1 monolayer (10-cm culture dish) from 6-well plate and let them grow until they cover the entire surface of OP9/DLL1. (A-E) Each bar in the images indicates 200 μm. (F) Almost all T cells induced from MAIT cell-derived iPSCs are MR1-tetramer positive and TCRβ^+^ cells (but not recognized by 6-FP-loaded MR1 tetramer).
  b. Prepare iPSC single cell suspension (see step 5 and 6 in before you begin).
  c. Count the cells and resuspend them at a concentration of 1.2 × 10^5^ cells/mL in Differentiation medium.
  d. Seed 1 mL of iPSC suspension per 10-cm OP9/DLL1 dish.
  e. Return the plates to the CO_2_ incubator.
2. Medium change (Day 3).
  a. Remove all the medium.
  b. Add 10 mL of Differentiation medium.
  c. Return the dishes to the CO_2_ incubator.
3. Harvesting mesodermal cells (Day 5, Figure 1B).
  a. Remove the medium from the dish in which rosebud-shaped cell clusters have formed.
  b. Wash the dish with 5 mL of PBS twice.
  c. Spread 2 mL of 0.25% Trypsin-1 mM EDTA evenly on the dish.
  d. Incubate the dish in a 5% CO_2_ incubator at 37°C for 3–5 min.
  e. Confirm that the cells are detached and add 8 mL of OP9 medium.
  f. Using a 1000-μL tip, disrupt the cell clumps by slightly vigorous but not bubbly pipetting (about 15 times).
  g. Place the dishes in the CO_2_ incubator for 45–60 min to re-adhere OP9/DLL1 cells onto the dish.
  h. Collect the medium containing floating cells with a 10-mL pipette and pass them through a 40 μm cell strainer.
  i. Centrifuge the cells at 400 × *g* for 5 min.
  j. Remove the supernatant and resuspend the cells in 10 mL of differentiation medium.
  k. Add 0.5 μL of 100 μg/mL FLT3-L per 10 mL of the cell suspension (final concentration 5 ng/mL).
  l. Prepare new OP9/DLL1 dish (see step 4 in before you begin) and remove the medium.
  m. Spread the cell suspension with FLT3-L to a new OP9/DLL1 dish.
  n. Return the dishes to the CO_2_ incubator.
4. Harvesting lymphoblasts (Day 8, Figure 1C).
  a. Take up the medium from the dish with a 10-mL pipette and use it to wash the surface with sufficient force not to break the OP9/DLL1 monolayer. Such pipetting allows semi-adherent blast cells to be detached from the OP9/DLL1 monolayer.
  b. Once sufficient pipetting has been completed, transfer the cell suspension into a 50-mL conical tube through a 40 μL cell strainer.
  c. Centrifuge the cells at 400 × *g* for 5 min.
  d. Remove the supernatant and resuspend the cells in 2 mL of reMAIT medium (stored at 4°C up to 10 days) per 10-cm dish.
  e. Seed 2 mL of cell suspension per well of a 6-well plate with the OP9/DLL1 monolayer.
5. Medium change (Day 10, 12, 14, Figure 1D).
  a. Remove all the medium.
  b. Add 2 mL of reMAIT medium per well.
  c. Return the plates to the CO_2_ incubator.
6. Expansion of reMAIT cells (Day 16 or later, Figure 1E).
  a. When the round, shiny cells grow to cover the OP9/DLL1 monolayer, remove them by pipetting with sufficient force not to break the OP9/DLL1 monolayer and collect the cell suspension into a 15-mL conical tube.
  b. Centrifuge the cells at 400 × *g* for 5 min.
  c. Remove the supernatant and resuspend the cells in 10 mL of reMAIT medium.
  d. Seed the cell suspension onto a new 10-cm OP9/DLL1 dish.
  e. Change the half of medium every other day or split the expanded cells into additional dishes.
7. Flow cytometry analysis for reMAIT cells (Figure 1F).
  a. Remove differentiated cells (reMAIT cells) from the OP9/DLL1 monolayer by pipetting.
  b. Replace 1 × 10^4^ – 5 × 10^4^ cells into a well of 96-well plate or a FACS tube.
  c. Wash the cells with FACS buffer (stored at 4°C up to 3 months).
  d. Resuspend the cells in the reaction mixture below containing the antibodies and MR1 tetramer for phenotyping.

**Table:** Reaction mixture

Reagent	Amount /5 × 10^4^ cells
APC MR1 tetramer /5-OP-RU or APC MR1 tetramer /6-FP	0.1 μL
PE/Cy7 TCRβ	0.5 μL
FACS buffer	50 μL
  e. Incubate at 20°C–25°C for 20 min.
  f. Wash the cells with FACS buffer.
  g. Resuspend the cells in FACS buffer containing 7-AAD (dead/live discrimination; dilution 1:200, stored at 4°C up to 6 months).
  h. Acquire the cells with a flow cytometer.
    **CRITICAL:** OP9/DLL1 dishes should be used within 4–5 days after preparation; if OP9/DLL1 dishes are older than 4–5 days, iPSCs may not adhere.
    **CRITICAL:** MAIT-iPSCs should not exceed 80% of the confluency during culture (before starting differentiation on OP9/DLL1). Overconfluent cells should not be used for differentiation step, as they show poor differentiation potential.
    ***Note:*** Morphological changes of the cells during differentiation are shown in Figures 1A–1E. Almost all T cells induced from MAIT-iPSCs are MR1-tetramer positive MAIT cells (Figure 1F). This is because MAIT-iPSCs harbor the MAIT cell-specific TCR gene rearrangement.^9^
    **Pause point:** reMAIT cells in the early logarithmic proliferative stage (normally on day 18–22) can be cryopreserved. Frozen reMAIT cells can be grown again on OP9/DLL1 dish with 20% FBS/αMEM containing IL-7 (reMAIT medium without FLT3-L) for further experiments.

### Preparation of mouse NK cells from C57BL/6

**Timing: 3 h**

Coexistence of MAIT and NK cells enhances the cytolytic activity against cancer cells more than each alone. Here the process of isolation and purification of NK cells from the mouse spleen is shown. Although isolation kits are available from various vendors, we use the BioLegend MojoSort Mouse NK cell isolation kit (BioLegend) combined with LS columns (Miltenyi Biotec).

8. Isolate the spleens from three euthanized 8- to 12-week-old C57BL/6 mice.
9. Place a 40 μm cell strainer in a 10 cm petri dish and place the spleens on the strainer.
10. Add 3–4 mL of cR10 and mush the spleens with a gasket portion of the plunger of a 2.5-mL syringe to strain out the cells.
11. Transfer the cell suspension into a 15-mL tube, rinse the petri dish with another 5 mL of cR10, and collect in a 15-mL tube.
12. Centrifuge at 400 × *g* for 4 min and aspirate the supernatant.
13. Add 3 mL of 67% Percoll (stored at 4°C up to 3 months) into a new 15-mL tube.
14. Suspend the cell pellet from step 12 with 8 mL of 40% Percoll (stored at 4°C up to 3 months).
15. Set the pipettor’s dispense speed to low and slowly layer the cell suspension in 40% Percoll above 3 mL of 67% Percoll with a 10-mL pipette.

***Note:*** At this time, the tube is tilted at an angle of about 30 degrees from the table surface and the cell suspension is added so that it flows down along the inner wall, forming a transparent layer.

16. Centrifuge at 400 × *g* for 20 min at 20°C–25°C with break-off.
17. Collect a white layer of cells at the boundary of the two Percoll layers into a new 15-mL tube.
18. Fill up the tube with PBS and mix well by inverting the tube.
19. Centrifuge at 700 × *g* for 7 min.
20. Aspirate the supernatant and suspend the cells in 10 mL of MACS buffer (stored at 4°C up to 3 months).
21. Filter the cell suspension with 40 μm cell strainer.
22. Count the cells. Usually, 3–5 × 10^7^ cells can be recovered from three spleens.
23. Adjust the cell concentration at 1 × 10^8^/mL with MACS buffer.
24. Add 2 μL of biotin antibody cocktail per 1 × 10^7^ cells.
25. Incubate on ice for 15 min.
26. Add 5 mL of MACS buffer and centrifuge at 400 × *g* for 5 min.
27. Suspend the cells in MACS buffer at 1 × 10^8^/mL.
28. Add 2 μL of streptavidin nanobeads per 1 × 10^7^ cells.
29. Incubate on ice for 15 min.
30. Add 5 mL of MACS buffer and centrifuge at 400 × *g* for 5 min.
31. Suspend the cells in 5 mL of MACS buffer.
32. Collect magnetic beads negative fraction using LS column.
  a. Place the LS column in a magnetic separator.
  b. Equilibrate the LS column with 3 mL of MACS buffer.
  c. Set an empty 15-mL conical tube under the LS column to collect the cell fraction that is not attached to the magnet (negative fraction).
  d. Apply the cell suspension from step 31 to the LS column through a 40 μm cell strainer or screen mesh and collect the fraction that passes through the LS column.
  e. After the liquid has completely entered the column, wash the LS column with 3 mL of MACS buffer, and collect it in the tube as well.
  f. Repeat step 32-e one more time.
33. Centrifuge the collected cell fraction at 400 × *g* for 5 min.
34. Suspend the cells with 3 mL of cR10.
35. Count the cells.
36. Take 1 × 10^4^ cells in a 96-well plate well or a FACS tube.
37. Add the following reaction mixture to check the purity of NK cells.

**Table:** Reaction mixture

Reagent	Amount /5 × 10^4^ cells
FITC NK1.1	0.5 μL
PE CD49b	0.5 μL
FACS buffer	50 μL

38. Incubate at 20–25°C for 20 min in the dark.
39. Wash the cells with FACS buffer.
40. Resuspend the cells in FACS buffer containing 7-AAD (dead/live discrimination).
41. Acquire the cells with a flow cytometer.

**Figure 2:**
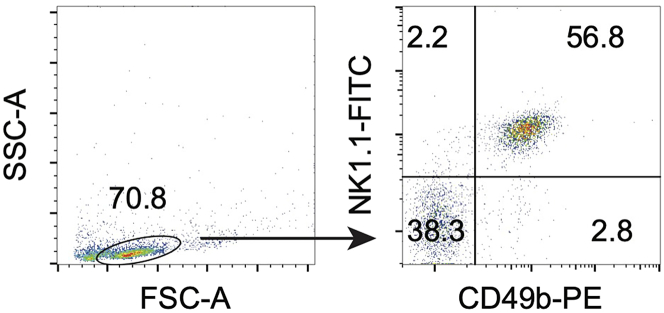
NK cell isolation from C57BL/6 spleen cells Mouse NK cells isolated by negative selection with magnetic microbeads were stained with NK1.1 and CD49b. Representative data are shown. The number in the quadrant shows the percentage of each subset.

***Note:*** An average of 1.5 × 10^8^ spleen cells can be obtained from three C57BL/6 mice, from which 1.5 to 5 × 10^6^ NK cells can be isolated. Flow cytometric analysis of the final product shows that approximately 50–60% are NK1.1^+^CD49b^+^ (Figure 2).

**CRITICAL:** Be sure to add 2 μL of biotin antibody cocktail per 1 × 10^7^ cells. Adding less antibody would compromise the purity of NK cells.

### Cytolytic activity of reMAIT cells against YAC-1

**Timing: 1 day**

Since YAC-1 is a lymphoma, it is difficult to distinguish it from MAIT cells, which are also T lymphocytes, on scatter plots in the flow cytometric analysis. Therefore, prior to co-culture with reMAIT cells, YAC-1 is fluorescently labeled by incorporating CFSE. After co-culture, the cells are stained with live/dead reagent. Flow cytometric analysis will reveal the cytolytic activity of reMAIT cells against YAC-1 by gating CSFE-labeled YAC-1 and measuring the percentage of dying or dead cells in YAC-1.

42. CFSE labeling of YAC-1.
  a. Prepare 1 × 10^6^ YAC-1.
  b. Centrifuge the cells at 400 × *g* for 5 min.
  c. Remove the supernatant.
  d. Suspend the cells in pre-warmed 1 mL of 1 μM CFSE in PBS.
  e. Incubate at 37°C for 15 min in a water bath.
  f. Add 10 mL of cR10.
  g. Centrifuge the cells at 400 × *g* for 5 min.
  h. Remove the supernatant.
  i. Suspend the cells in 10 mL of cR10.
  j. Incubate at 37°C for 30 min in CO_2_ incubator.
  k. Centrifuge the cells at 400 × *g* for 5 min.
  l. Remove the supernatant.
  m. Suspend the cells in 5 mL of cR10 (final cell concentration at 2 × 10^5^ cells/mL).
43. Co-culture of CFSE-labeled YAC-1 and reMAIT cells.
  a. Prepare 1.4 × 10^6^ of reMAIT cells.
  b. Centrifuge the cells at 400 × *g* for 5 min.
  c. Remove the supernatant.
  d. Resuspend the cells in 700 μL of cR10 (final concentration at 2 × 10^6^ cells/mL).
  e. Make the following dilution of reMAIT cells.

**Table:** 

	6 × 10^5^ cells/mL reMAIT cells	2 × 10^5^ cells/mL reMAIT cells
2 × 10^6^ cells/mL reMAIT	150 μL	50 μL
cR10	350 μL	450 μL
  f. Prepare 700 μL of 2 × 10^6^ cells/mL NK cells in cR10 by using the NK cell suspension from step 34.
  g. Make the following dilution of NK cells.

**Table:** 

	6 × 10^5^ cells/mL NK cells	2 × 10^5^ cells/mL NK cells
2 × 10^6^ cells/mL NK cells	150 μL	50 μL
cR10	350 μL	450 μL
  h. Plate CFSE-labeled YAC-1 (T:Target), reMAIT cells and NK cells (E:Effector) in triplicate in a 96-well V-bottom plate as shown in Table 1.

**Table 1:** The amounts of each subset of the cells required for cytolytic assay against Yac-1

	E/T ratio	CFSE labeled YAC-1 (2 × 10^5^ cells/mL)	reMAIT			NK			cR10
			2 × 10^6^ cells/mL	6 × 10^5^ cells/mL	2 × 10^5^ cells/mL	2 × 10^6^ cells/mL	6 × 10^5^ cells/mL	2 × 10^5^ cells/mL	
YAC-1 vs. reMAIT	10	50 μL (1 × 10^4^ cells)	50 μL (1 × 10^5^ cells)	-	-	-	-	-	100 μL
	3	50 μL (1 × 10^4^ cells)	-	50 μL (3 × 10^4^ cells)	-	-	-	-	100 μL
	1	50 μL (1 × 10^4^ cells)	-	-	50 μL (1 × 10^4^ cells)	-	-	-	100 μL
YAC-1 vs. NK	10	50 μL (1 × 10^4^ cells)	-	-	-	50 μL (1 × 10^5^ cells)	-	-	100 μL
	3	50 μL (1 × 10^4^ cells)	-	-	-	-	50 μL (3 × 10^4^ cells)	-	100 μL
	1	50 μL (1 × 10^4^ cells)	-	-	-	-	-	50 μL (1 × 10^4^ cells)	100 μL
YAC-1 vs. reMAIT+NK	10	50 μL (1 × 10^4^ cells)	50 μL (1 × 10^5^ cells)	-	-	50 μL (1 × 10^5^ cells)	-	-	50 μL
	3	50 μL (1 × 10^4^ cells)	-	50 μL (3 × 10^4^ cells)	-	-	50 μL (3 × 10^4^ cells)	-	50 μL
	1	50 μL (1 × 10^4^ cells)	-	-	50 μL (1 × 10^4^ cells)	-	-	50 μL (1 × 10^4^ cells)	50 μL
No-effector control	-	50 μL (1 × 10^4^ cells)	-	-	-	-	-	-	150 μL
  i. Incubate for 4 h at 37°C in a CO_2_ incubator.
44. Live/dead staining using Zombie dye.
  a. Dilute Zombie Violet in PBS at 1:500.
  b. After 4 h incubation, centrifuge the 96-well plate at 500 × *g* for 5 min in a plate centrifuge.
  c. Dump the supernatant by quickly inverting and flicking the plate.
  d. Add 100 μL of diluted Zombie Violet into each well.
  e. Incubate at 20-25°C for 15 min, shielded from light.
  f. Add 100 μL of FACS buffer and centrifuge at 500 × *g* for 5 min.
  g. Dump the supernatant by quickly inverting and flicking the plate. Scratch the bottom of the plate to loosen the cell pellets.
  h. Add 200 μL of FACS buffer and centrifuge at 500 × *g* for 5 min.
  i. Dump the supernatant by quickly inverting and flicking the plate. Scratch the bottom of the plate to loosen the cell pellets.
  j. Add 100 μL of FACS buffer.
  k. Acquire the cells with a flow cytometer.
45. Analyze the flow cytometric data (Figure 3).

**Figure 3:**
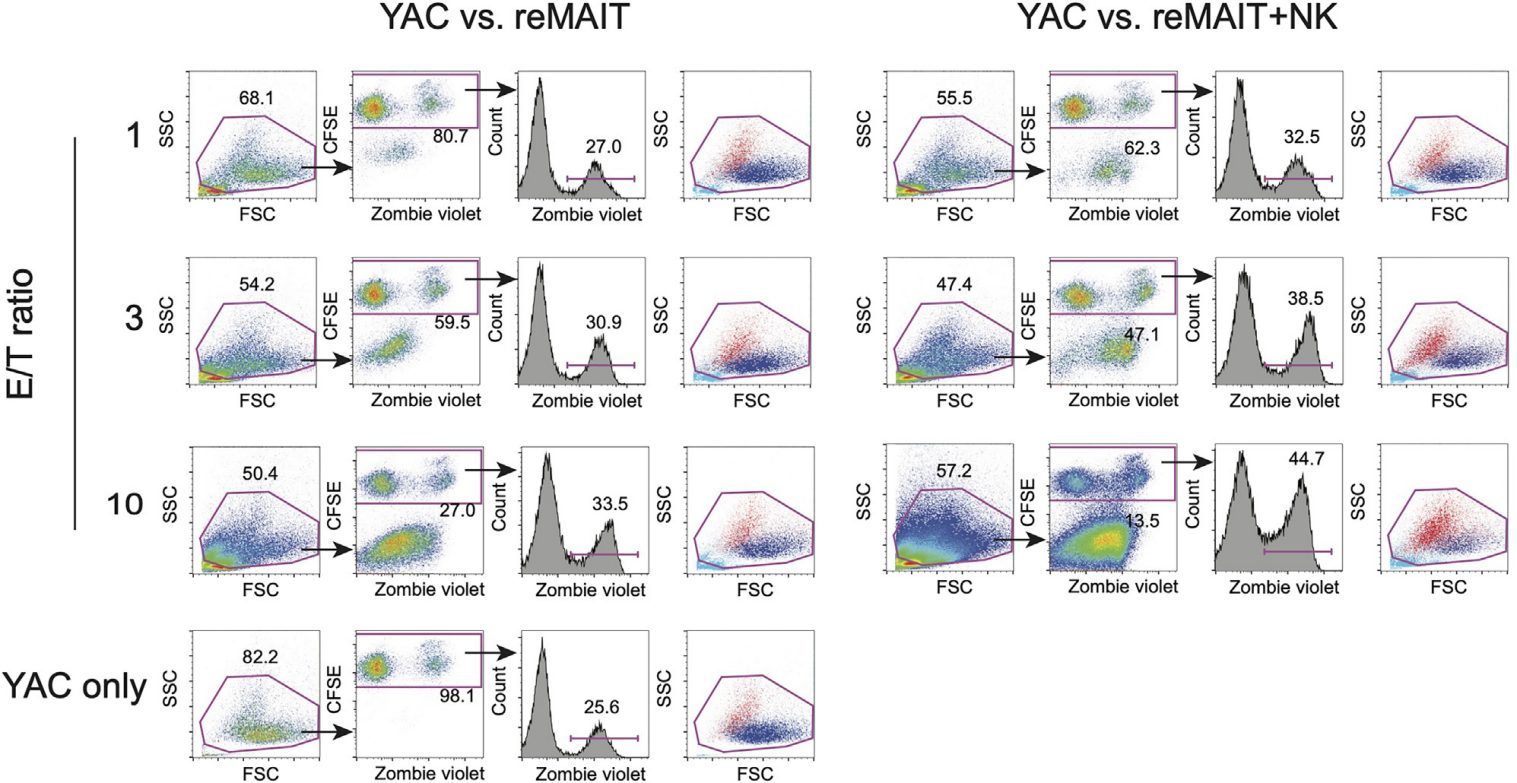
Gating strategy for measuring cytolytic activity of reMAIT cells (left panel) and reMAIT cells plus NK cells (right panel) against YAC-1(YAC) Most of the cells other than debris are gated in the scatter plots (leftmost columns). YAC-1 is detected as CFSE-positive (second columns from left); cells seen as CSFE-negative are the effector cells (reMAIT cells and/or NK cells). The percentage of Zombie positive cells (considered as dying or dead cells) among CFSE positive YAC-1 is shown as a histogram (second columns from right). Dead (or dying) YAC-1 (shown in red) are located in the diagonal on the scatter plots. Live YAC-1 is shown in blue. The cells indicated in light blue are CFSE-negative cells including the effector cells (reMAIT and NK) (rightmost columns).
46. Calculate “percent lysis” using the following formula: {(% of Zombie^+^ cells among target cells in the presence of effector cells) - (% of Zombie^+^ cells among target cells in the absence of effector cells)}/ {100 − (% of Zombie^+^ cells among target cells in the absence of effector cells)}/100.

***Note:*** Because YAC-1 culture contains many dead cells, a significant number of Zombie^+^ cells are detected even in the absence of effector cells (high background).

**CRITICAL:** Be sure to use 1 μM CFSE. The use of more or less concentrated CFSE would make it difficult to identify the CSFE-positive cells within the FITC-channel in flow cytometry.

### Cytolytic activity of reMAIT cells against LLC

**Timing: 3 days**

LLC is adherent and is generally resistant to killing by cytolytic CD8^+^ T cells and NK cells. We found that reMAIT cells can kill LLC by co-culturing for 16–20 h using the following strategies.

47. Plating of LLC (Day 0).
  a. Trypsinize semi-confluent LLC and prepare 1 × 10^5^ cells/mL cell suspension in 8% FBS/DMEM.
  b. Spread 100 μL per well of the cell suspension into a 96-well flat-bottom culture plate (1 × 10^4^ cells/well).
  c. Incubate at 37°C for 16–20 h in CO_2_ incubator.
48. Co-culture of LLC and reMAIT cells and/or NK cells (Day 1).
  a. Prepare 3 × 10^6^ of reMAIT cells.
  b. Centrifuge the cells at 400 × *g* for 5 min.
  c. Remove the supernatant.
  d. Resuspend the cells in 1 mL of cR10 (final concentration at 3 × 10^6^ cells/mL).
  e. Make the following dilution for reMAIT cells.

**Table:** 

	1 × 10^6^ cells/mL reMAIT cells	3 × 10^5^ cells/mL reMAIT cells	1 × 10^5^ cells/mL reMAIT cells
3 × 10^6^ cells/mL reMAIT	200 μL	60 μL	20 μL
cR10	400 μL	540 μL	580 μL
  f. Prepare 1 mL of 3 × 10^6^ cells/mL NK cells in cR10 by using the NK cell suspension from step 34.
  g. Make the following dilution of NK cells.

**Table:** 

	1 × 10^6^ cells/mL NK cells	3 × 10^5^ cells/mL NK cells	1 × 10^5^ cells/mL NK cells
3 × 10^6^ cells/mL NK cells	200 μL	60 μL	20 μL
cR10	400 μL	540 μL	580 μL
  h. Remove the medium from each well of 96-well plate of LLC culture (see step 47).
  i. Add appropriate amounts of reMAIT cells in a 96-well plate seeded with LLC in triplicate as shown in Table 2.

**Table 2:** The amounts of each subset of the cells required for cytolytic assay against LLC

	E/T ratio	reMAIT				NK				cR10
		3 × 10^6^ cells/mL	1 × 10^6^ cells/mL	3 × 10^5^ cells/mL	1 × 10^5^ cells/mL	3 × 10^6^ cells/mL	1 × 10^6^ cells/mL	3 × 10^5^ cells/mL	1 × 10^5^ cells/mL	
LLC vs. reMAIT	30	100 μL (3 × 10^5^ cells)	-	-	-	-	-	-	-	100 μL
	10	-	100 μL (1 × 10^5^ cells)	-	-	-	-	-	-	100 μL
	3	-	-	100 μL (3 × 10^4^ cells)	-	-	-	-	-	100 μL
	1	-	-	-	100 μL (1 × 10^4^ cells)	-	-	-	-	100 μL
LLC vs. NK	30	-	-	-	-	100 μL (3 × 10^5^ cells)	-	-	-	100 μL
	10	-	-	-	-	-	100 μL (1 × 10^5^ cells)	-	-	100 μL
	3	-	-	-	-	-	-	100 μL (3 × 10^4^ cells)	-	100 μL
	1	-	-	-	-	-	-	-	100 μL (1 × 10^4^ cells)	100 μL
LLC vs. reMAIT+NK	30	100 μL (3 × 10^5^ cells)	-	-	-	100 μL (3 × 10^5^ cells)	-	-	-	-
	10	-	100 μL (1 × 10^5^ cells)	-	-	-	100 μL (1 × 10^5^ cells)	-	-	-
	3	-	-	100 μL (3 × 10^4^ cells)	-	-	-	100 μL (3 × 10^4^ cells)	-	-
	1	-	-	-	100 μL (1 × 10^4^ cells)	-	-	-	100 μL (1 × 10^4^ cells)	-
No-effector control	-	-	-	-	-	-	-	-	-	200 μL
  j. Incubate for 16–20 h at 37°C in a CO_2_ incubator.
49. Live/dead staining using Zombie dye (Day 2).
  a. Dilute Zombie Violet in PBS at 1:1000.
  b. Aspirate the medium from a 96-well plate.
  c. Wash the wells with 200 μL of PBS.
  d. Add 30 μL of the diluted Zombie Violet solution into each well.
  e. Incubate at 20°C–25°C for 15 min, shielded from light.
  f. Wash the wells with 200 μL of PBS twice.
  g. Add 30 μL of 0.25% Trypsin-1 mM EDTA.
  h. Incubate at 37°C for 5 min.
  i. Add 70 μL of 10% FBS/DMEM.
  j. Acquire the cells with a flow cytometer.
50. Analyze the flow cytometric data (Figure 4).

**Figure 5:**
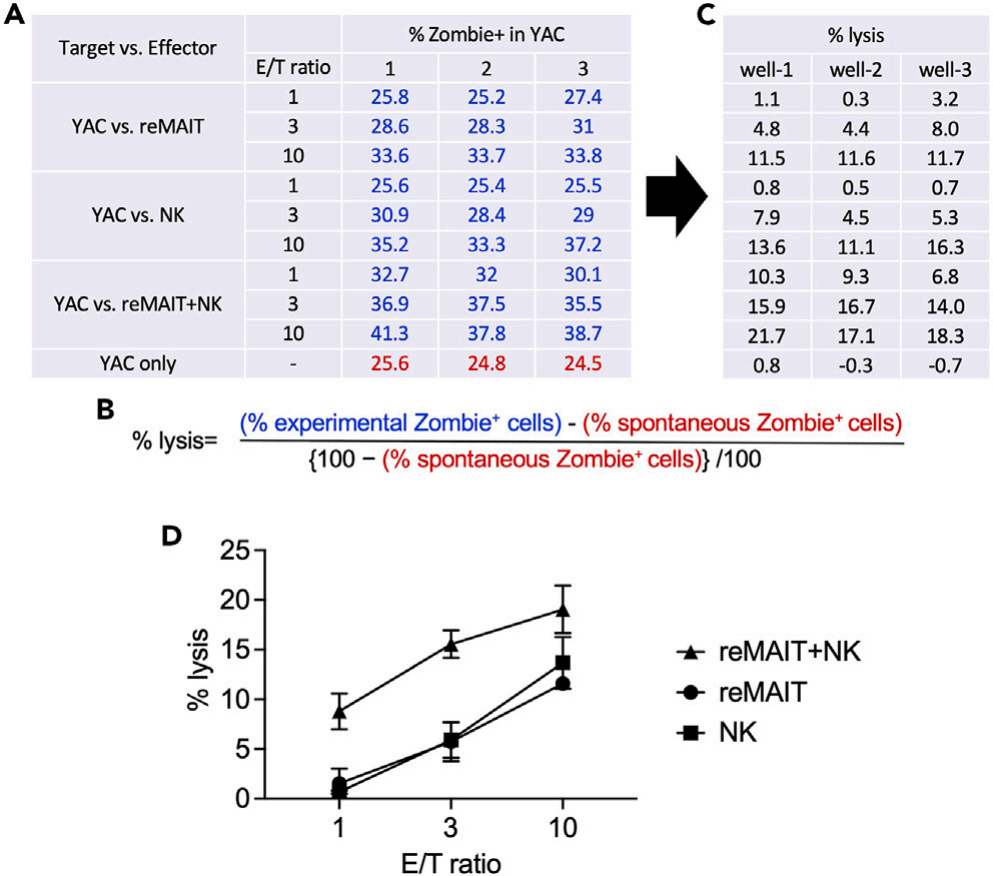
Anti-YAC-1 cytolytic activity of reMAIT cells and NK cells (A) Example data of % Zombie^+^ in YAC-1 according to the gating shown in Figure 3. The percentages of Zombie^+^ cells among YAC-1 in the presence of effector cells (experimental Zombie^+^ cells) are shown in blue, and the percentages of Zombie^+^ cells among YAC-1 in the absence of effector cells (spontaneous Zombie^+^ cells) are shown in red. (B) The formula for calculating the percentage of lysis. (C) The percentages of lysis calculated by applying the values in (A) to the formula (B). (D) Cytolytic activity of reMAIT cells (reMAIT ●), NK cells (NK ◼), and NK cells plus reMAIT cells (NK + reMAIT ▲). Data (C) are plotted (% lysis vs. E/T ratio) as mean ± SD.
51. Calculate “percent lysis” using the following formula: {(% of Zombie^+^ cells among target cells in the presence of effector cells) - (% of Zombie^+^ cells among target cells in the absence of effector cells)}/ {100 − (% of Zombie^+^ cells among target cells in the absence of effector cells)}/100.

***Note:*** NK cells alone would not lyse LLC.

**CRITICAL:** Be sure to incubate LLC with reMAIT cells for 16–20 h. Less incubation time would compromise the lytic activity of reMAIT cells.

## Expected outcomes

### Cytolytic activity of reMAIT cells against YAC-1

In general, the cytolytic activity of reMAIT cells and that of NK cells against Yac-1 is almost-identical at the indicated E/T ratio. At E/T = 10, the percent lysis by reMAIT cells or NK cells is ∼12%, which is further enhanced by the combination (reMAIT cells + NK cells) (see Figure 5D).

**Figure 4:**
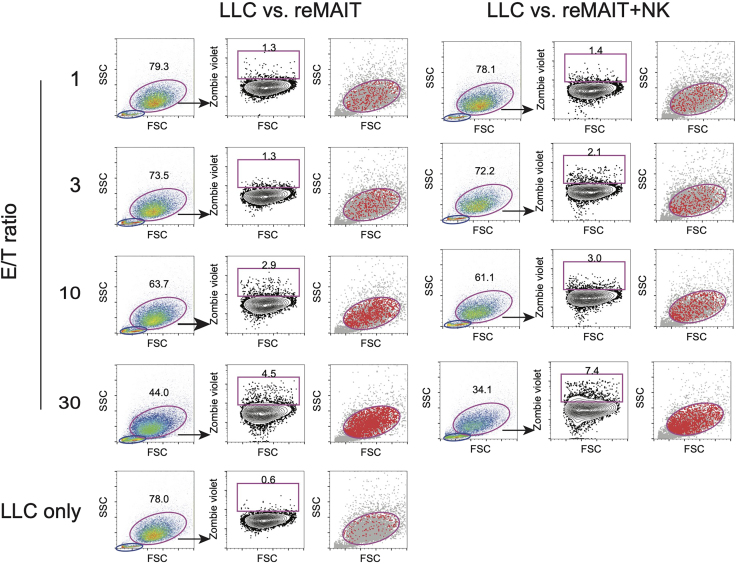
Gating strategy for measuring LLC killing activity of reMAIT cells (left panel) and reMAIT cells plus NK cells (right panel) SSC high/FSC high cells (circled in pink) are gated as LLC in the scatter plots. Effector cells (reMAIT and/or NK cells) are shown as SSC low/FSC low (circled in blue) in the scatter plots (left columns). Zombie positive cells among LLC are considered to be dead or dying. The number indicates the percentage of Zombie positive cells in LLC (middle columns). Dead or dying cells are shown in red in the scatter plot (right columns, gated for LLC).

### Cytolytic activity of reMAIT cells against LLC

**Figure 6:**
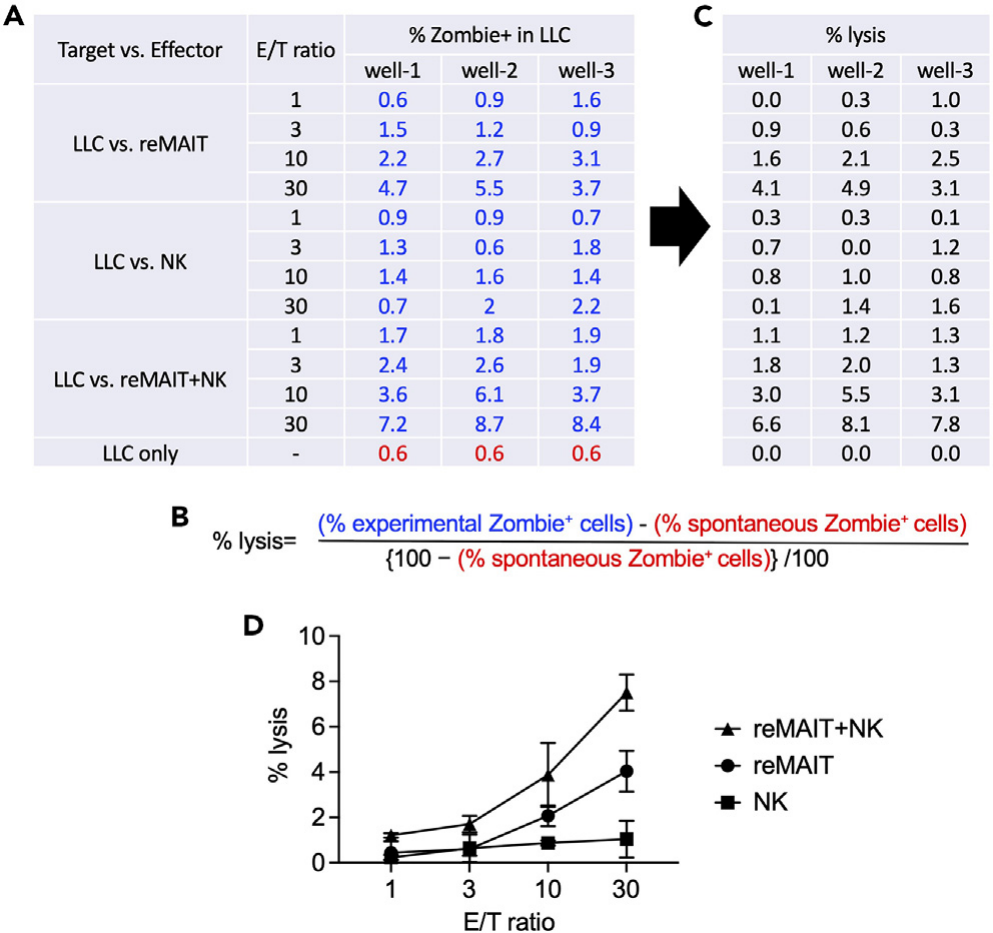
Cytolytic activity of reMAIT cells and NK cells against LLC (A) Example data of % Zombie^+^ in LLC according to the gating shown in Figure 4. The percentages of Zombie^+^ cells among LLC in the presence of the effector cells (experimental Zombie^+^ cells) are shown in blue, and the percentages of Zombie^+^ cells among LLC in the absence of the effector cells (spontaneous Zombie^+^ cells) are shown in red. (B and C) (B) The formula for calculating the percentage of lysis (C) The percentages of lysis calculated by applying the values in (A) to the formula (B). (D) Cytolytic activity of reMAIT cells (reMAIT ●), NK cells (NK ◼), and NK cells plus reMAIT cells (NK + reMAIT ▲). Data (C) are plotted (%lysis vs. E/T ratio) as mean ± SD.

In general, NK cells cannot kill LLC, whilst reMAIT cells can, albeit to a lesser extent than Yac-1. At E/T = 30, the percent lysis by reMAIT cell is ∼4%. Moreover, when NK cells and reMAIT cells are used together, it reaches ∼7% (see Figure 6D).

## Limitations

Killing activity of reMAIT cells and NK cells against Yac-1 could be observed after 4 h of culture. Such short-term killing activity is likely dependent on the release of granzymes and perforin, but the exact mechanism of killing is currently unknown.

Furthermore, 16–20 h incubation with reMAIT cells is required to confirm killing activity against LLC. This indicates that another pathway, such as death ligand-mediated killing, is involved, but the pathway responsible for the activity has yet to be identified. Furthermore, since it takes more than 36 h to observe killing activity after LLC seeding, LLC has proliferated almost 4-fold by the time of measurement. This may mask the true killing ability of reMAIT cells. In such cases, time lapse microscopy and/or Incucyte Immune cell killing assays may be needed.

There might be concern that aspiration of the medium after co-culturing with LLCs in step 49b will remove dead LLCs. As noted above, time-lapse observations may confirm whether more time is needed for cells to be completely lysed and detached. However, this protocol is based on the premise that the damaged cells will remain adherent even after the medium is removed. However, if other adherent cells are to be tested or if this protocol is to be applied to the cytotoxic assay of CTLs, it is recommended that the culture supernatant be taken separately, centrifuged to precipitate the detached cells, and stained for dead/live detection together with trypsin-treated cells.

While reMAIT cells seem to exert cytolytic activity against a wide range of tumor cell lines, we have confirmed just two lines. For application of the present assay to other tumor cell lines, optimal incubation time and the requirement of fluorescent labeling should be determined empirically.

## Troubleshooting

### Problem 1

Loss of differentiation supporting ability of OP9/DLL1 (Step 3–4 in before you begin, step 1–7 in step-by-step method details).

### Potential solution

After several rounds of reMAIT cell redifferentiation with the same lot of OP9/DLL1 (generally 20 passages or more), lymphoblast formation and/or lymphoblast proliferation becomes defective. Change the lot of OP9/DLL1. Keep the pace of passage constant (3 days).

### Problem 2

Redifferentiation into reMAIT cells does not work even if used proper OP9/DLL1 (Step 3–6 in before you begin, step 1–7 in step-by-step method details).

### Potential solution

Successful redifferentiation entirely depends on the FBS lot. Perform the lot check before starting the whole experiments. It is useful to check the proliferation of OP9/DLL1 cells and the ability to support differentiation of MAIT- iPSCs into the lymphoblast (up to day 8 of differentiation). Gibco-FBS may be a first choice with relatively little lot variation; even Value FBS is compatible. Currently, we are using Value FBS-Brazil (cat# 10270).

### Problem 3

The proliferation of LLC is poor when passaged (Step 8 in before you begin).

Expansion of LLC is highly dependent on the cell density.

### Potential solution

LLC is passaged twice a week. If proliferation is still poor, add 1/3 to 1/2 volumes of the conditioned medium (supernatant from the previous LLC culture) to the culture.

### Problem 4

It is difficult to distinguish between dead and live LLC after Zombie staining at 1:1000 dilution (step 49 in step-by-step method details).

### Potential solution

While we routinely use 1:1000 dilution of Zombie, 1:500-1:300 dilution could be used. However, more concentrated Zombie solution results in high background, which may compromise the interpretation.

### Problem 5

The purity of NK cells is rarely superior to 70% (steps 8–41, step-by-step method details).

### Potential solution

If purer NK cells are required, cell sorting with NK1.1 and CD49b antibody should be performed.

However, in such a case, more mice are necessary to obtain the sufficient amounts of NK cells.

## Resource availability

### Lead contact

Further information and requests for resources and reagents should be directed to and will be fulfilled by the lead contact, Hiroshi Wakao (hwakao@dokkyomed.ac.jp).

### Materials availability

The mouse MAIT cell-derived iPSC clones used in this study will be made available to qualified investigators by having Material Transfer Agreement.

## Data Availability

This study did not generate datasets or codes.
